# ASSOCIATIONS BETWEEN COVID-19 PERCEPTIONS AND PREVENTIVE BEHAVIORS IN DEMENTIA CAREGIVING DYADS

**DOI:** 10.1093/geroni/igac059.476

**Published:** 2022-12-20

**Authors:** Jiaming Liang, Maria Aranda, Yuri Jang

**Affiliations:** University of Southern California, Los Angeles, California, United States; University of Southern California, Los Angeles, California, United States; University of Southern California, Los Angeles, California, United States

## Abstract

This study adopted a dyadic perspective to examine how the perceptions of COVID-19 (i.e., anxiousness & hopefulness) of dementia caregiving dyads are associated with their engagement in personal (e.g., washing hands, wearing mask) and social (e.g., avoiding physical contact and going restaurants/bars) preventive behaviors. Multiple cross-sectional Actor-Partner Interdependence Models (APIMs) were estimated using data from the 2020 NHATS/NSOC COVID-19 Supplements (N=1565). In the anxiousness models, participants’ own feeling of anxiousness was associated with their own engagement in personal preventive behaviors (actor effects), and the perceived anxiousness of PLWD was associated with personal preventive behaviors of caregivers (partner effect). In the model on social preventive behaviors, both actor and partner effects were found on dementia caregiving dyads. No effect was found in the models on hopefulness. Our findings extend understandings of mutual influence within the caregiving dyads and demonstrate the possibility of developing interventions for caregivers to promote PLWD’s health behaviors.

